# Exploring the role of orexins in the modulation of social reward

**DOI:** 10.1007/s00213-024-06688-5

**Published:** 2024-09-20

**Authors:** Inês M. Amaral, Sara Ouaidat, Laura Scheffauer, Anna E. Granza, Diogo G. Monteiro, Ahmad Salti, Alex Hofer, Rana El Rawas

**Affiliations:** 1 Division of Psychiatry I, Department of Psychiatry, Psychotherapy, Psychosomatics and Medical Psychology, Medical University Innsbruck, Innsbruck, 6020 Austria; 2 University Clinic of Ophthalmology and Optometry, Kepler University Hospital, Johannes Kepler University Linz, Linz, 4020 Austria

**Keywords:** Social interaction, Reward, Conditioned place preference, Orexin, Stress, Depression, SB334867, Orexin 1 receptor, Social preference, Social impairments

## Abstract

**Rationale:**

positive social interactions are essential for mental health, by offering emotional support, reducing stress levels, and promoting resilience against drugs of abuse effects. However, not all individuals perceive social interaction as rewarding.

**Objectives:**

the goal of this study was to investigate whether the modulation of the orexin system can shift passive coping and non-social behavior (vulnerable) to active coping and social behavior (resilient). This knowledge is primordial for stress- and addiction-related disorders, and for other psychiatric disorders involving impairment in social interaction.

**Methods:**

male C57/BL6N mice categorized into social and non-social groups, received injections of SB334867, a selective orexin 1 receptor (OX1R) antagonist, before the conditioning sessions with a male conspecific of the same weight and age.

**Results:**

our results from the conditioned place preference test (CPP) show that SB334867 has no effect on social preference in non-social mice, but it reduces their stress levels and depression-like behavior. These effects appear to be due to a higher OX1R expression in the basolateral amygdala (BLA), a stress-related brain area, of non-social mice compared to their social counterparts.

**Conclusions:**

these data suggest that the orexin system may be a target to alleviate stress and depression-like behavior in non-social individuals rather than to promote social reward.

**Supplementary Information:**

The online version contains supplementary material available at 10.1007/s00213-024-06688-5.

## Introduction

Impairment of social interaction (SI) is a hallmark of many mental disorders (Timmermans and Schilbach 2014). Interestingly, SI reward has been shown to have beneficial effects (El Rawas and Saria 2016), such as reducing stress levels (Lemos et al. 2021) and promoting resilience against drugs’ effects (El Rawas et al. 2020). However, some individuals show a lack of interest in social interactions (Kasanova et al. 2018) and do not perceive SI as rewarding (Granza et al. 2023).

In rodents, SI reward can be evaluated using the conditioned place preference paradigm (CPP) (Bardo and Bevins 2000). Generally, social CPP is assessed by placing the animals in one chamber of the CPP with an assigned social partner for half of the conditioning sessions and alone in the opposite chamber of the CPP for the other half of the sessions (El Rawas and Saria 2016). Animals that spend more time in the social-paired chamber during the CPP test express a preference for SI. While both mice and rats can express a preference for SI reward, our previous studies have shown that mice are less likely to express social CPP compared to rats (Granza et al. 2023). Indeed, whereas 85% of rats express SI reward, only 61 to 62% of C57Bl/6J mice showed social CPP (Granza et al. 2023; Lemos et al. 2021). The question at hand is why SI is perceived as more rewarding by certain mice than by others, and what the neurological basis of this social or non-social phenotype is. Furthermore, the question arises as to what extent it is possible to transform the non-social phenotype into a social one. The importance of these questions resides in the fact that positive SI helps to lessen mental health symptoms (Acoba 2024). Therefore, finding neurobiological correlates that can shift passive coping and non-social behavior (vulnerable) to an active coping and social behavior (resilient) is primordial not only for stress and addiction-related disorders, but also for other mental disorders involving impairment in SI.

This study investigated whether the orexin system is a possible candidate in this respect. Orexin A, also called hypocretin 1 (OXA/HCRT1), and orexin B or hypocretin 2 (OXB/HCRT2), are neuropeptides synthesized in neurons originating in the lateral hypothalamus (LH) that project throughout the brain and bind to two widely expressed G-protein coupled receptors, the orexin-1 receptor (OX1R) and the orexin-2 receptor (OX2R) (Ouaidat et al. 2024; Sakurai et al. 1998; Sun et al. 2021). While the OX1R shows selective binding affinity for OXA, the OX2R has an equal affinity for both neuropeptides (Sakurai et al. 1998; Sun et al. 2021). Among other functions, this system has been shown to be implicated in stress (Berridge and España 2005; Grafe et al. 2018) and reward (Aston-Jones et al. 2009; Borgland et al. 2010; DiLeone et al. 2003; Harris et al. 2005; Muschamp et al. 2014). Indeed, optogenetic stimulation of orexin neurons in the LH regulates corticosterone release and a variety of behaviors and physiological hallmarks of the stress response (Bonnavion et al. 2015). In addition, photostimulation of orexin neurons decreases the time spent in SI, suggesting that stimulation of the orexin system increases the anxiety state of an animal (Heydendael et al. 2014). It was also found that lower orexin expression is associated with active coping strategies, therefore suggesting that low levels of orexins may be a biomarker to predict resilience to stress (Grafe et al. 2018). Indeed, inhibition of orexin neurons during social defeat stress produces an increase in SI and a decrease in depressive-like behaviors in passively coping rats (Grafe et al. 2018). In addition, another study found that lower orexin function may be indicative of resilience, rather than vulnerability (Chung et al. 2014). When OXA was centrally administered, it decreased the expression of social play behavior (Reppucci et al. 2020), considered as the highest rewarding component in SI (Vanderschuren et al. 2016). In the same study, it was also shown that central blockade of OX1R increased social play in low baseline social play subjects (Reppucci et al. 2020).

The current study investigated the effect of OX1R antagonism on the expression of social CPP. We hypothesized that systemic administration of an OX1R antagonist would help shift the non-social and passive coping behavior of C57BL/6 N mice to social and active coping behavior. In addition, we investigated how OX1R antagonism affects the stress profile of social and non-social mice. Finally, OX1R expression was compared between social and non-social mice in stress-related brain areas: the basolateral amygdala (BLA), the central amygdala (CeA), the bed nucleus of the stria terminalis (BNST), and the paraventricular thalamus (PVT).

## Materials and methods

### Animals

Male C57BL/6 N mice at young adulthood (6 weeks old) were ordered from Janvier Labs (Le Genest-Saint-Isle, France) and singly housed upon arrival. Given that isolated animals display a high motivation for SI as a result of social isolation (Trezza et al. 2009), animals were kept in their home cages for at least 10 days prior to the start of the behavioral experiments and remained isolated during the experiments. Mice were housed under controlled environmental conditions (temperature ~ 18–23 °C and humidity ~ 40–60%) with water and food supplied *ad libitum*. All behavioral experiments were conducted during the light phase (between 8 a.m. and 5 p.m.) of the 12-hour light/dark schedule. Ethical consent was obtained from the Austrian National Animal Experiment Ethics Committee (BMWF 2021 − 0.308.024).

### OX1R antagonist (SB334867) solution

SB334867 (#A12786B002, Adooq Bioscience, Irvine, USA) was prepared at a final concentration of 3 mg/mL by dissolving it in a saline solution containing 30% Hydropropyl-β-Cyclodextrin (HP-β-CD) (Sigma Aldrich). In brief, HP-β-CD was dissolved in a 0.9% saline solution and heated in a warm bath for effective dissolution. SB334867 was added once the solution became clear, and the resulting solution was then sonicated for up to 45 min. A 30% HP-β-CD in saline solution was used as a vehicle (control) solution.

### Conditioned Place Preference (CPP)

#### CPP Apparatus

A three-chambered CPP apparatus (64 cm wide × 32 cm deep × 31 cm high) made of unplasticized polyvinylchloride was used for the behavioral experiments. The middle (neutral) compartment (10 × 30 × 30 cm) with white walls and a white floor led to the two conditioning compartments (25 × 30 × 30 cm) through two removable doors. The two conditioning compartments had walls with distinct features (black and white horizontal or vertical stripes) as well as different stainless steel floors (168 holes with a diameter of 0.5 cm or 56 slits (4.2 × 0.2 cm)). Following each session, the CPP chambers were cleaned using a 70% camphorated ethanol solution. Behavior was recorded using a video camera and the analysis was done offline via ANY-maze Video Tracking software (Stoelting Europe, Dublin, Ireland).

#### SI CPP

SI CPP consisted of 3 main steps:

- Pretest (day 1): During the pretest, mice were placed in the middle compartment for 15 s before they were allowed to explore the entire apparatus for 30 min. Analysis of the time spent in each compartment during the pretest was used to determine the natural preference of each animal.
- Conditioning (days 2–5): Conditioning comprised 4 training days with 2 sessions per day (either with a social partner or alone, each lasting 30 min), one in the morning and the other in the afternoon, separated by 4 h. During the SI conditioning sessions, each mouse was paired with a weight-matched conspecific and placed in the less preferred compartment (determined in the pretest). Social partners were assigned based on weight and compartment preference and remained the same throughout the entire experiment. Mice were placed alone (no stimulus) in the opposite compartment (preferred compartment during the pretest). During conditioning sessions, mice were confined to the compartment where they were placed, as access to the middle compartment was blocked by a door.
- Test 1 (day 6): During the test, mice were once again allowed to explore the entire apparatus for 30 min. The expression of CPP of each mouse was determined by calculating the social preference score (PS), which is the time spent in the social-paired compartment during the test minus the time spent in the same compartment during the pretest, with a cut-off equal to 0 (i.e., PS < 0 = non-social phenotype, PS > 0 = social phenotype).

#### OX1R blockade

d. Conditioning (days 7–10): Conditioning was conducted as previously described (see SI CPP section, b). The assigned pairs of mice received the same treatment: either an intraperitoneal (i.p.) injection of the OX1R antagonist solution (30 mg/kg) or the vehicle solution (30% HP-β-CD in 0.9% saline solution) 30 min before each SI conditioning session. The treatment choice of each pair was chosen randomly. The dose of SB334867 was selected based on previous studies (Rodgers et al. 2001; Smith et al. 2009).
e. Test 2 (day 11): A second CPP test was performed in accordance with the first one (see SI CPP section, c) and the PS of each animal was calculated again as described above.

### Forced swim test

A forced swim test (FST) was performed on the final day (day 12). Mice were placed for 6 min in a cylindrical glass tank (40 cm high, 15 cm wide), half filled with water (temperature ~ 23–25 °C), and their movements were recorded using a video camera. The FST was preceded by either an i.p. injection of the OX1R antagonist solution (30 mg/kg) or vehicle, 30 min before the test. At the end, mice were sacrificed using an overdose of sodium pentobarbital (i.p. 2 mL/kg, Release ^®^ 300 mg/mL). An observer blind to the experimental groups manually calculated the total time mice spent immobile.

### Incorrect transitions of cephalocaudal grooming

Grooming is an important element of rodent behavior with a pattern of cephalocaudal progression (paw licking→nose/face wash→body wash→tail/genitals wash). When this transition is performed incorrectly, the incorrect transitions of cephalocaudal progression between different grooming patterns can be used as a stress marker in rats (Kalueff and Tuohimaa 2005). The grooming stages are defined as no grooming (0), paw licking (1), nose/face/head wash (2), body grooming (3), leg licking (4), and tail/genitals grooming (5). Correct transitions between grooming stages include the following progressive transitions: 0–1, 1–2, 2–3, 3–4, 4–5, and 5 − 0. Four main types of incorrect transitions have been described: they include aborted, prematurely terminated (e.g., 3 − 0 and 4 − 0), skipped (e.g., 1–5 and 2–5), reversed (e.g., 3 − 2, 4 − 1, and 5 − 2), and incorrectly initiated (e.g., 0–4 and 0–5). In the present study, the percentage of incorrect transitions of cephalocaudal grooming (Amaral et al. 2021; Lemos et al. 2021) was evaluated manually during the second CPP test (T2) by an observer blind to the experimental conditions.

### Quantitative real-time PCR

Animals were sacrificed 30 min after the test by an overdose of sodium pentobarbital (i.p., 2 mL/kg, 300 mg/mL). Brains were removed, immediately frozen at -40 °C using isopentane then stored at -80 °C. BNST brain tissue was punched out of thaw-mounted coronal 100-µm sections at -15 °C in a cryostat (Leica CM3050 S) using a sample corer (Fine Science Tools, Foster City, CA, USA, 15 G). Total RNA was isolated from the dissected brain regions using the Monarch^®^ Total RNA Miniprep Kit (#T2010G, New England BioLabs, Ipswich, MA, USA) according to the manufacturer’s protocol. RNA purity and concentration were evaluated using a NanoDrop spectrophotometer (peqlab Biotechnologies, Erlangen, Germany). In total, 200 ng of RNA was reverse transcribed into cDNA using the OneScript^®^ Plus cDNA Synthesis Kit from Applied Biological Materials Inc. Richmond, BC, Canada (abm) in a total volume of 20 µL. After dilution with 80 µL of water, 5 µL of the diluted cDNA were used as templates for amplification (duplicates) using the SsoAdvanced Universal SYBR^®^ Green Supermix (Bio–Rad Laboratories, Hercules, CA, USA). Real-time polymerase chain reaction quantification (qRT-PCR) was performed on a CFX96 Touch Real-Time PCR Detection System (Bio–Rad Laboratories) using the following cycle settings: 30 s at 95 °C, 5 s at 95 °C (40 cycles), and 30 s at 60 °C. All PCR primers were designed using the PrimerSelect 7.1.0 software (DNASTAR Lasergene). The efficiency of the primers was verified using a 2-fold serial dilution of cDNA and melt-curve analysis. The cycle threshold (Ct) and ΔCt values were calculated using CFX Maestro™ software (version 2.2) with GAPDH as a reference gene. Relative changes in gene expression (fold change) were determined through the 2 ^− ΔΔCt^ method. The primers used in this study are detailed in Table 1.

**Table 1:** List of primers used to assess the relative expression of corticotropin-releasing factor (CRF) using Gapdh as an internal normalization reference

Primers	Forward (5′–3′)	Reverse (5′–3′)
Gapdh	AGGGCTCATGACCACAGTC	CAGCTCTGGGATGACCTTG
CRF	TCGGCTGTCCCCCAACTC	CTGCAGCAACACGCGGAAAA

### Perfusion fixation and brain preparation

For the immunohistochemistry experiments, mice were sacrificed by anesthesia (i.p. sodium-pentobarbital 2 mL/kg) at the end of the CPP test in order to undergo perfusion surgery using 4% paraformaldehyde (PFA) in 0.1 M phosphate-buffered saline (PBS) for fixation. After transcardiac perfusion, mice were decapitated and their brains were removed to be immersed in 4% PFA in 0.1 M PBS for 24 h at 4 °C. Afterwards, the brains were immersed in a 30% sucrose in 0.1 M PBS solution for cryoprotection. After approximately 48 h, the brains were frozen in ice-cold isopentane (-40 °C) and then stored at -80 °C until further use.

### Immunohistochemistry

40 µM coronal brain sections from the brain areas of interest were collected using a cryostat and then stored in Assorter buffer (0.25 M TBS, 0.01% NaN3, pH 7.4) at 4 °C until further processing. The brain sections were first washed 3 times for 10 min in 1x Tris buffered saline (TBS) at room temperature (RT). After this step, permeabilization was performed using a 0.3% Triton-X-100 in 1x TBS solution, 2 times for 5 min. The sections were placed in blocking solution for 1 h at RT and then incubated overnight at 4 °C with the primary antibody diluted in blocking solution (anti-OX1R rabbit polyclonal antibody, #OAR-001, 1:800, Alomone labs). The following day, the sections were washed in 1x TBS, 3 times for 10 min, and then incubated for 2 h at RT in the dark with the Alexa Fluor-conjugated secondary antibody diluted in blocking solution (Alexa Fluor 555 goat anti-rabbit, Invitrogen, Thermo Fisher Scientific Inc, #A-21428, 1:1000). A final washing step was performed with 1x TBS, 3 times for 10 min. The sections were then mounted on gelatin-coated slides, with a DAPI-containing mounting medium (ROTI^®^Mount FluorCare DAPI, Roth). Glass slides were covered with a Menzel-Glaser coverslip and allowed to dry overnight before being stored in the dark at 4 °C to prevent immunofluorescence fading. The sections were scanned using a Zeiss fluorescence microscope set at ×40 magnification equipped with a camera (Axioplan 2 Imaging) interfaced to a PC. The counting of the positive nuclei in the images was conducted offline using the Fiji^®^ Software (ImageJ) cell counter plugin in a blind condition. The mean of three sections per animal was used in the statistical analysis.

### Statistical analysis

All data were analyzed using GraphPad Prism (version 10). Results are presented as mean ± standard error of the mean (SEM). Differences between groups were assessed either by a two-tailed unpaired Student’s *t*-test or two- or three-way ANOVA followed by Šidák’s multiple comparisons test. Correlation between two variables was also evaluated. Bonferroni multiple testing correction was performed for the immunohistochemistry data in case of a statistically significant result. P-values < 0.05 were regarded as statistically significant.

## Results

### Social and non-social mice had comparable baseline stress levels

**Fig. 1:**
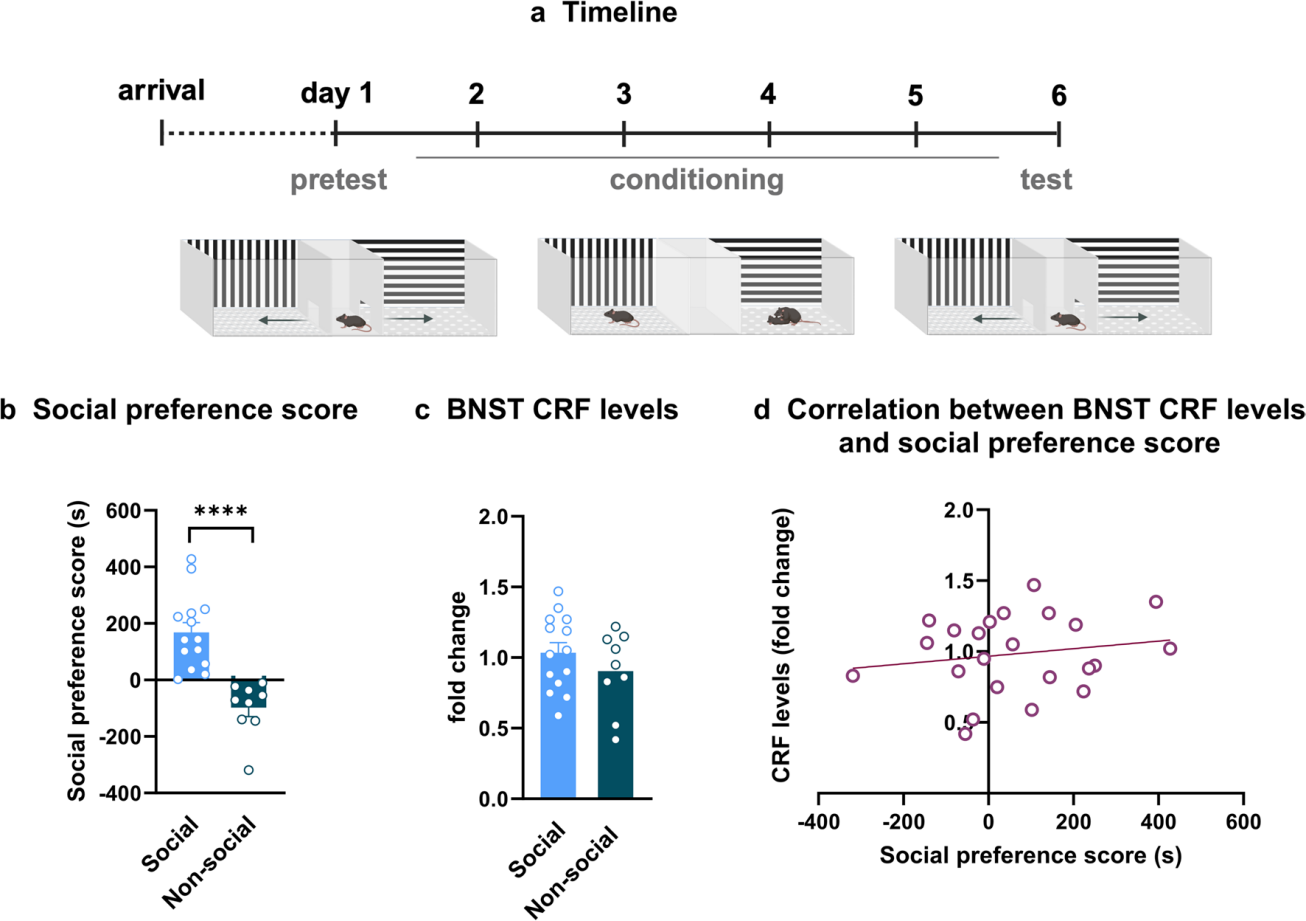
CRF levels in the BNST were similar in social and non-social mice. (a) Timeline. (b) SI CPP scores were used for segregating mice into social and non-social groups. Student’s t-test **** *p* < 0.0001, *n* = 9–14. (c) CRF levels in the BNST in social and non-social mice. Student’s t-test, *p* > 0.05, *n* = 9–14. (d) Correlation between social preference score and CRF levels in the BNST. *p* > 0.05

Mice were conditioned to SI with a partner for four days in one compartment and were placed “alone” in the other compartment before being tested for expression of social CPP (Fig. 1a). After segregating the mice into social and non-social groups based on their preference score in the CPP test [unpaired two-tailed Student’s *t*-test, social vs. non-social, t (21) = 5.245; *p* < 0.0001 – Fig. 1b], CRF levels in the BNST were evaluated to investigate whether baseline stress levels would differ between the opposite social profiles. CRF levels in the BNST were similar in social and non-social mice (unpaired two-tailed Student’s *t*-test, social vs. non-social, t (21) = 1.125; *p* = 0.2731 - Fig. 1c). Additionally, no correlation was found between CRF expression in the BNST and the preference score of mice (*p* = 0.4335 - Fig. 1d).

### Inhibition of OX1R failed to shift the non-social phenotypes of mice

**Fig. 2:**
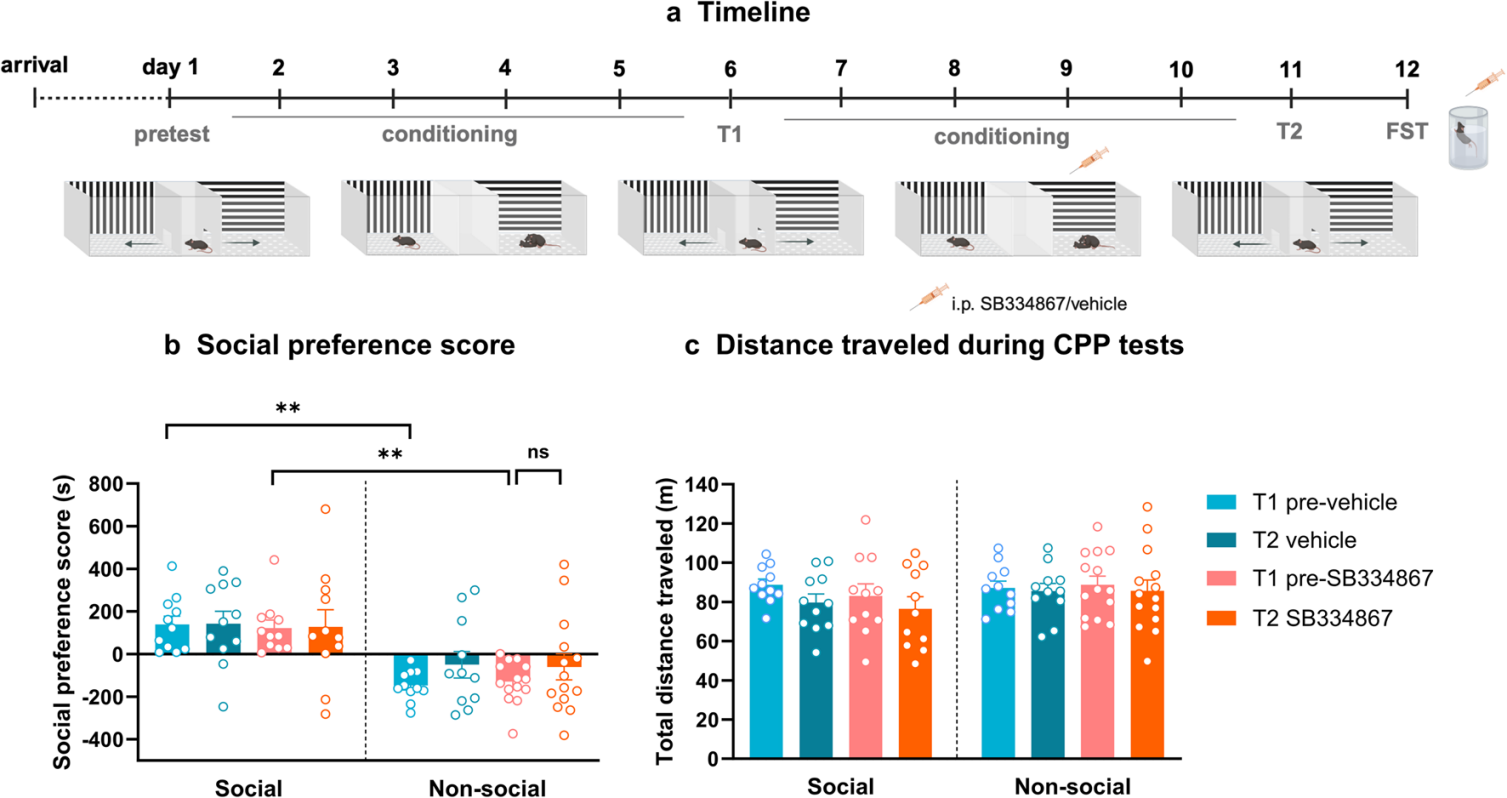
OX1R antagonist (SB334867) did not shift the non-social phenotypes of mice. (a) Timeline. (b) Social preference score of social and non-social mice during the first (T1) and the second (T2) conditioned place preference test (i.e., before/after treatment). (c) Total distance traveled by the distinct groups during T1 and T2. Three-way ANOVA, followed by Šídák’s multiple comparisons test, ** *p* < 0.01, ns = non-significant, *n* = 11–14. FST = forced swim test, i.p = intraperitoneal

In this experiment, we investigated the effects of the orexin 1 receptor antagonist, SB334867, on social reward. After segregation into social and non-social profiles, based on the social preference score obtained in the CPP test 1 (T1), mice were randomly assigned to receive either SB334867 or a vehicle solution. The administration of SB334867 or the vehicle was performed 30 min prior to each SI conditioning session, with each mouse receiving a total of four i.p. injections. Following the conditioning period, mice were tested for social CPP in a second test (T2) (Fig. 2a).

At T1, social and non-social mice had different social phenotypes [three-way ANOVA, social phenotype effect, F (1, 43) = 33.13, *p* < 0.0001; treatment effect, F (1, 43) = 0.02299, *p* = 0.8802; test time effect, F (1, 43) = 1.753; *p* = 0.1925; social phenotype x treatment, F (1, 43) = 0.06163, *p* = 0.8051; social phenotype x test, F (1, 43) = 1.408, *p* = 0.2419; treatment x test, F (1, 43) = 0.03528, *p* = 0.8519; social phenotype x treatment x test time, F (1, 43) = 0.05141, *p* = 0.8217; Šídák’s multiple comparisons test: at T1 social pre-vehicle vs. non-social pre-vehicle, *p* < 0.01 and social pre-antagonist vs. non-social pre-antagonist, *p* < 0.01)]. At T2, social mice receiving vehicle injections during social conditioning remained in their social profile (Šídák’s multiple comparisons test, T1 pre-vehicle vs. T2 vehicle, *p* > 0.05). Furthermore, social mice receiving SB334867 kept their social profile (Šídák’s multiple comparisons test, T1 pre-antagonist vs. T2 antagonist, *p* > 0.05). Interestingly, from T1 to T2, four social mice reversed their profiles to non-social. Non-social mice receiving vehicle also kept their profile (Šídák’s multiple comparisons test, T1 pre-vehicle vs. T2 vehicle, *p* > 0.05). Three of these mice reversed their profiles from non-social to social. Contrary to expectations, treatment with the OX1R antagonist failed to shift the profile of non-social to social mice (Šídák’s multiple comparisons test: non-social: T1 pre-antagonist vs. T2 antagonist, *p* > 0.05). Indeed, only four mice out of fourteen shifted their profile from non-social to social after OX1R antagonist treatment (Fig. 2b).

In order to check for any possible effects of SB334867 on locomotion, the total distance traveled during T1 and T2 was evaluated in all groups of mice. A three-way ANOVA test revealed that treatment with OX1R antagonist yielded no effects on locomotion [treatment effect, F (1, 43) = 0.1674, *p* = 0.6844; social phenotype effect, F (1, 43) = 1.205, *p* = 0.2785; test time effect, F (1, 43) = 5.166, *p* = 0.0281; social phenotype x treatment, F (1, 43) = 0.3744, *p* = 0.5438; social phenotype x test time, F (1,43) = 1.424, *p* = 0.2392; treatment x test time, F (1, 43) = 0.01683, *p* = 0.8974; social phenotype x treatment x test time, F (1, 43) = 0.207; *p* = 0.6514] (Fig. 2c).

### OX1R antagonist (SB334867) attenuated stress levels in non-social mice

**Fig. 3:**
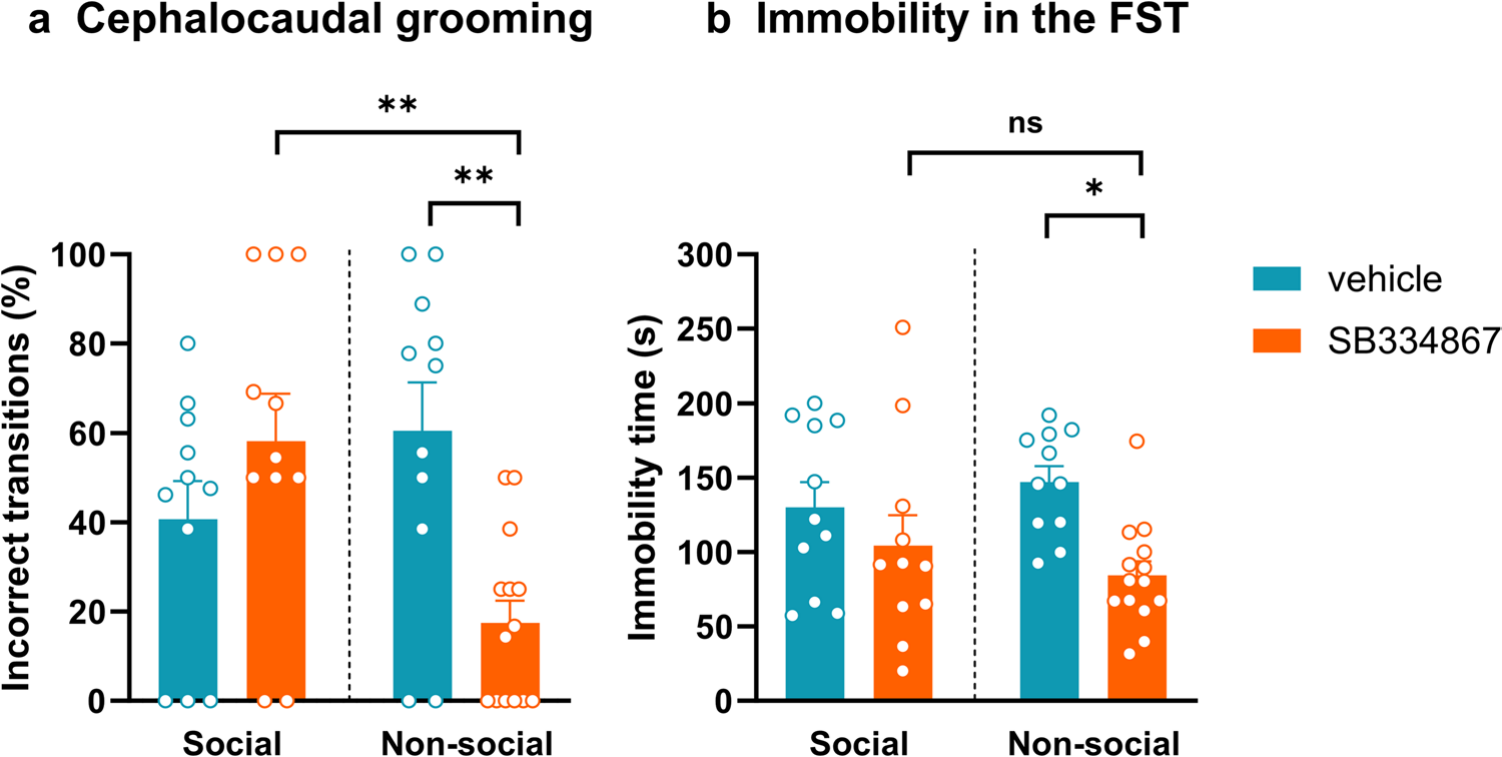
OX1R blockade reduced stress levels only in non-social mice. (a) Percentage of incorrect transitions of cephalocaudal grooming during the second conditioned place preference test (T2). (b) Time spent immobile during the FST. Two-way ANOVA, followed by Šídák’s multiple comparisons test, * *p* < 0.05, ** *p* < 0.01, ns = non-significant, *n* = 11–14

To evaluate the effects of OX1R antagonist injections on stress, we assessed the percentage of incorrect transitions of cephalocaudal grooming in social and non-social mice at T2 (in a treatment-free state). In social mice, treatment with SB334867 did not alter the percentage of incorrect transitions of cephalocaudal grooming [two-way-ANOVA, treatment effect, F (1, 43) = 1.447; *p* = 0.2355, social phenotype effect, F (1, 43) = 2.156; *p* = 0.1493, treatment x social phenotype, F (1, 43) = 12.12; *p* = 0.0012; Šídák’s multiple comparisons test, social vehicle vs. antagonist, *p* > 0.05]. In non-social mice, SB334867 treatment significantly decreased the percentage of incorrect transitions of cephalocaudal grooming compared to vehicle-treated non-social mice (Šídák’s multiple comparisons test, non-social vehicle vs. antagonist, *p* < 0.01) and antagonist-treated social mice (Šídák’s multiple comparisons test, social antagonist vs. non-social antagonist, *p* < 0.01) (Fig. 3a).

One day after the second CPP (T2), mice underwent FST preceded by an injection of SB334867 or vehicle. In social mice, the immobility time in the FST was not affected by OX1R antagonist treatment [two-way ANOVA, treatment effect, F (1, 43) = 0.01109; *p* = 0.9166; social phenotype effect, F (1, 43) = 9.253; *p* = 0.0040; treatment x social phenotype, F (1, 43) = 1.624; *p* = 0.2094; Šídák’s multiple comparisons test, social vehicle vs. antagonist, *p* > 0.05]. In non-social mice, SB334867 treatment significantly reduced the immobility time compared to non-social mice that received vehicle (Šídák’s multiple comparisons test, non-social vehicle vs. antagonist, *p* < 0.05) (Fig. 3b). Yet, social mice and non-social mice receiving the antagonist treatment showed a comparable immobility time in the FST (Šídák’s multiple comparisons test, social antagonist vs. non-social antagonist, *p* > 0.05).

### OX1R expression in the BLA, a stress-related area, was higher in the non-social phenotype

**Fig. 4:**
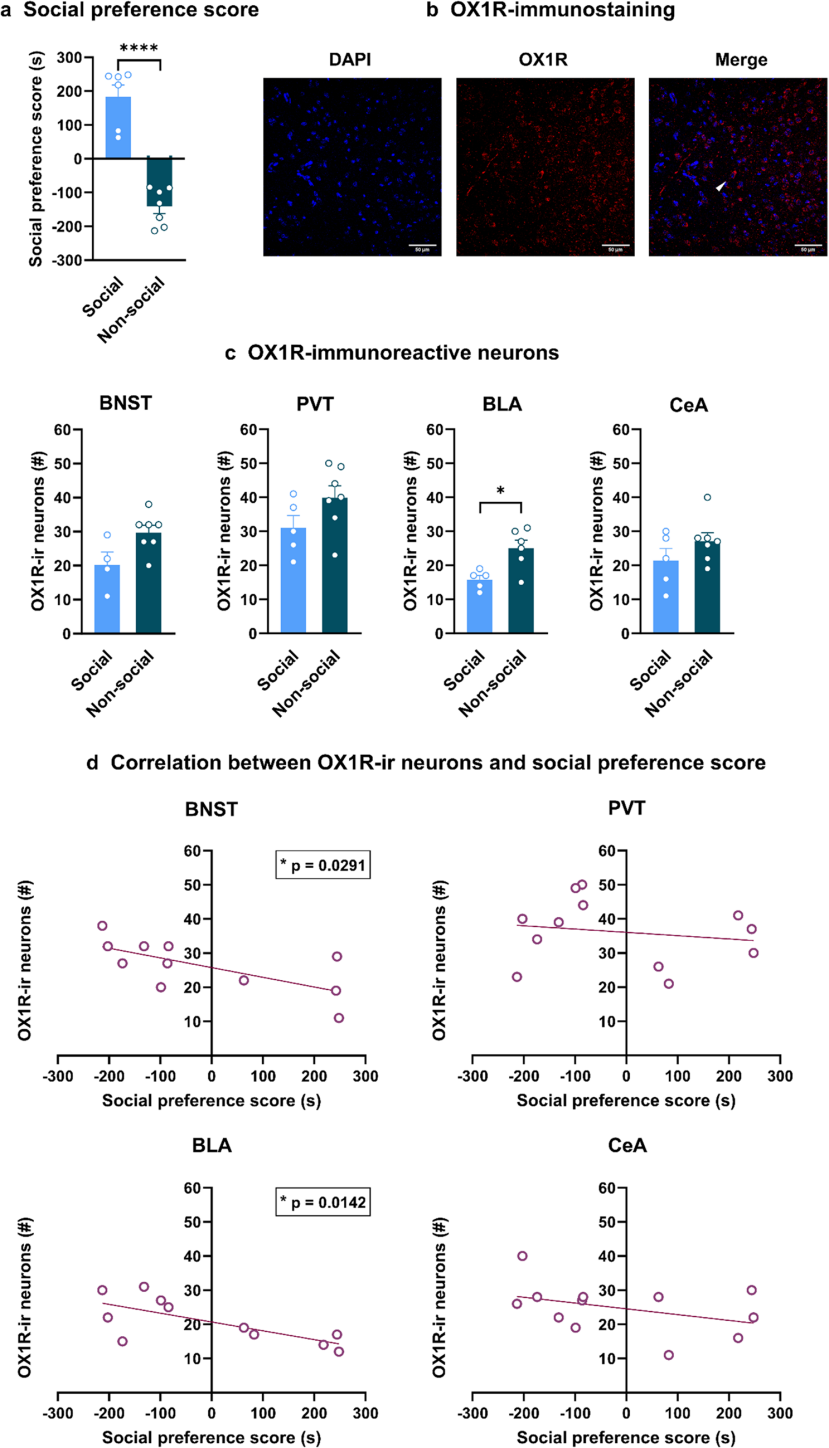
Non-social mice express a significantly higher number of OX1R-immunoreactive neurons in the BLA. (a) Social preference scores, used to categorize the mice according to their social profiles. Student’s t-test, **** *p* < 0.0001. *n* = 6–7. (b) Representative image of OX1R-immunoreactive neurons (arrow); blue: DAPI staining (nuclei); red: OX1R. Scale bar: 50 μm (c) OX1R-immunoreactive neurons in social and non-social mice in stress-related areas of the brain, including the BNST, the PVT, the BLA, and the CeA. Student’s t-test, followed by Bonferroni correction. * *p* < 0.05, *n* = 4–7. (d) Correlation between social preference score and number of OX1R-immunoreactive neurons in the different stress-related brain regions. * *p* < 0.05

In order to explore whether the selective effects of SB334867 treatment on stress-associated measures in non-social mice were due to different OX1R expression between social and non-social mice, OX1R-immunoreactive neurons were quantified in stress-related brain areas (supplementary Fig. 1) after being categorized based on their social preference [Two-tailed unpaired Student’s *t*-test, social vs. non-social: *p* < 0.0001, t (11) = 8.218] (Fig. 4a).

Non-social mice showed significantly higher OX1R expression in the BLA [Two-tailed unpaired Student’s *t*-test, social vs. non-social: t (9) = 3.185, *p* = 0.0111] but not in the CeA [Two-tailed unpaired Student’s *t*-test, social vs. non-social: t (10) = 1.365, *p* = 0.2021], in comparison to social mice. The difference in the BLA was maintained even after the application of Bonferroni correction (*p* < 0.05). Moreover, in the BNST, the expression of OX1R was higher in non-social mice compared to their counterparts [Two-tailed unpaired Student’s *t*-test, social vs. non-social: t (9) = 2.387, *p* = 0.0408]. However, after Bonferroni correction was applied, the result turned out to be non-significant (*p* = 0.1632). No significant difference in OX1R expression was observed in the PVT between social and non-social mice (Two-tailed unpaired Student’s *t*-test, social vs. non-social: t (10) = 1.707, *p* = 0.1185; ns) (Fig. 4b and c).

To further investigate whether any relationship exists between the social phenotype (i.e., preference score - PS) and the expression of OX1R, a correlation analysis was performed. A negative correlation between the PS and the number of OX1R-immunoreactive neurons was observed in the BLA (*p* = 0.0142, *r* = -0.7109) and the BNST (*p* = 0.0209, *r* = -0.6815). No correlation was found between the social preference score and the expression of OX1R in the CeA (*p* = 0.2013, *r* = -0.3970) or PVT (*p* = 0.5839, *r* = -0.1762) (Fig. 4d).

## Discussion

As a main finding, this study shows that OX1R antagonism failed to shift the non-social preference to a social one but could selectively decrease stress levels in non-social mice. This decrease appears to be associated with higher expression of OX1R found in the BLA, a brain region implicated in stress. Indeed, a negative correlation was found between OX1R-immunoreactive neurons in the BNST and BLA and the social preference score. Therefore, higher OX1R expression in these regions is linked to a lower preference for SI. Moreover, similar baseline stress levels were observed in social and non-social mice. Nevertheless, CRF levels in the BNST evaluated in social and non-social mice were not compared to a naïve control mice group.

In order to identify the social profile of mice, we conditioned them with a social partner of the same sex, age, and weight and then categorized these mice into social and non-social profiles based on their preference to spend time with this social partner. We hypothesized that treatment with SB334867 would shift the preference of non-social mice, but it seems that OX1R antagonism did not alter the rewarding effects of SI. Our findings contradict (Reppucci et al. 2020), who reported the involvement of the orexin system in the expression of social play behavior in juvenile rats. In their study, the first analyses revealed that central administration of the OX1R antagonist SB334867 did not alter the percentage of time juvenile rats engaged in social play (Reppucci et al. 2020). However, when categorized based on their baseline levels of social play, SB-334,867 differentially affected juvenile rats (Reppucci et al. 2020). Indeed, SB334867 treatment significantly increased social play expression in subjects with low baseline levels of social play and decreased social play expression in subjects with high baseline levels of social play (Reppucci et al. 2020). Another study also found that SB334867 administration reduced the time of SI with strangers in male mice (Dawson et al. 2023). Our findings show that SB334867 does not alter the preference of subjects with no preference for SI. One possible explanation is that, despite the highly rewarding effect of social play (Trezza et al. 2010), social preference is a measure of SI behavior that is not modulated by the orexin system. Indeed, orexin levels in the LH have been shown to be comparable in male mice with social and non-social profiles (Granza et al. 2023). Furthermore, no correlation has been detected between the social preference score and the orexin levels in the LH of mice (Granza et al. 2023). In line with these findings, the number of orexin-immunoreactive neurons has been reported to be similar in the LH of rats exposed or not exposed to social play (Reppucci et al. 2020). Yet, juvenile rats exposed to social play had a significantly greater Fos induction within orexin neurons compared to juvenile rats in the no social play condition (Reppucci et al. 2020). Moreover, male orexin-deficient mice displayed normal sociability compared to their wild-type littermates (Faesel et al. 2021). Interestingly, in this study, when comparing social preference scores at T1 and T2, four social mice (two vehicle-treated and two antagonist-treated) reversed their profile to non-social. This change could be due to the possible development of an aversion to the SI-associated compartment. Indeed, each SI conditioning session performed after T1 was preceded by an i.p. injection of either vehicle or the OX1R antagonist. These injections might have increased their p-p38 MAPK levels (Salti et al. 2015), thereby possibly promoting an aversive effect (Bruchas et al. 2007) associated with the social compartment. On the contrary, seven non-social mice (three vehicle-treated and four antagonist-treated) switched their preference from non-social to a social profile. Possibly, these mice required a greater number of SI conditioning sessions in order to express a social preference. Nevertheless, the social profile switch of the four non-social antagonist-treated mice is unlikely due to the effects of the SB334867 injections, as ten non-social mice clearly retained their non-social preference despite the increased number of social conditioning sessions. In fact, studies have shown that social preference or pro-sociality is relatively stable (Böhm et al. 2021; Carlsson et al. 2014), which reinforces the urge to explore a possible approach to shift the preference from non-social to social.

Since stress disrupts the general pattern of self-grooming’s uninterrupted cephalocaudal progression (Lemos et al. 2021), the percentage of incorrect transitions between different grooming patterns can be used as a behavioral marker of stress in rodents (Kalueff and Tuohimaa 2005). During the second social CPP test (T2), the percentage of incorrect cephalocaudal transitions was selectively reduced in non-social mice with prior OX1R antagonist treatment. Although this analysis was performed in treatment-free conditions, another study has reported that i.p. injections of SB334867 at a dose of 10 mg/kg led to a total blockade of OXA-induced increase in grooming time in rats, emphasizing the role of OX1R in grooming behavior elicited by OXA (Duxon et al. 2001). Furthermore, SB334867 reduced the total immobility time during the FST selectively in non-social mice when injected 30 min before the test. In accordance with our results, it has previously been shown that systemic treatment with SB334867 resulted in a reduction in behavioral despair, characterized by a decrease in immobility time in the FST (Alijanpour et al. 2019; Scott et al. 2011). However, in another study, pharmacological blockade of both OX1R and OX2R via bilateral microinjection of TCS1102, a potent dual orexin receptor antagonist, into the ventral pallidum (VP), led to a significant increase in immobility time in the FST (Ji et al. 2019). This behavior seems to be dependent on the orexin receptor subtypes involved, as OX1R and OX2R differentially modulate depression-like behavior (Scott et al. 2011). Indeed, the disruption of OX1R decreases behavioral despair, while the disruption of OX2R increases this behavior (Scott et al. 2011). Nevertheless, knocking down the expression of OX1R and OX2R separately in the VP significantly prolonged the immobility time of rats in the FST (Ji et al. 2019). These results suggest that the involvement of orexin receptors is region-specific, with the blockade of endogenous orexinergic inputs specifically in the VP resulting in depressive-like behaviors (Ji et al. 2019). In line with region specificity, a positive correlation has previously been found between OX1R mRNA expression in the amygdala and depressive behavior assessed by immobility time in the FST (Arendt et al. 2013). Nevertheless, central orexinergic system disruption through genetic or pharmacological manipulations that selectively target OX1R or OX2R results in opposite modulation of depression-like behavior (Scott et al. 2011). Our results also show that the effects of SB334867 on stress and despair-like behaviors only occur in non-social mice. In order to investigate the cause of this selectivity, OX1R expression levels were evaluated in stress-associated brain areas. Increased levels of OX1R in the BLA in non-social mice, compared to social mice, may be the reason for the selective effects of SB334867 on the percentage of incorrect cephalocaudal grooming transitions during the CPP test and immobility time in the FST. Moreover, we detected a negative correlation between the levels of OX1R in the BLA and the BNST and social preference. This means that the higher the OX1R expression is in these stress-associated brain regions, the lower the social preference, thereby amplifying the effects of OX1R antagonism in non-social mice on stress-related measures.

Different strains of mice vary in their expression of social reward. Indeed, upon employing a social CPP procedure, it was demonstrated that social proximity is rewarding for juvenile mice from three inbred strains (A/J, C57BL/6J and DBA/2J), while mice from a fourth strain (BALB/cJ) are much less responsive to social contact (Panksepp and Lahvis 2007). When comparing CD1 to C57BL/6J female mice, CD1 but not C57BL/6J mice demonstrated robust social CPP (Ramsey et al. 2022). Indeed, adult female C57BL/6J mice expressed significantly less social reward than males from the same strain (Granza et al. 2023). In this study, we used the C57BL/6 N male mice as they develop preference to SI to a lesser extent than C57BL/6J male mice (Pinheiro et al. 2016). Consequently, the administration of OX1R antagonist would have noticeably resulted in a shift in their behavior from non-social to social phenotypes. One limitation of this study is that mice were categorized into social and non-social groups based only on their social preference score. Performing additional tests that measure different additional aspects of social interaction would have provided a more accurate categorization between social and non-social mice.

In conclusion, OX1R treatment did not shift the social profile of non-social mice but decreased stress and despair-like behavior selectively in these mice. These data suggest that the orexin system may be a target to alleviate stress and depression in non-social individuals rather than to promote social reward.

## Supplementary Information

Below is the link to the electronic supplementary material.

ESM1 (DOCX 569 KB)
