# Supplementary material for: Exploring the role of orexins in the modulation of social reward
Source: Psychopharmacology (Berl). 2024 Sep 20;242(2):401–12. doi: 10.1007/s00213-024-06688-5 (PMC11775052; doi:10.1007/s00213-024-06688-5)
Supplement: Supplementary file 1 — (DOCX 569 KB) [file 213_2024_6688_MOESM1_ESM.docx]

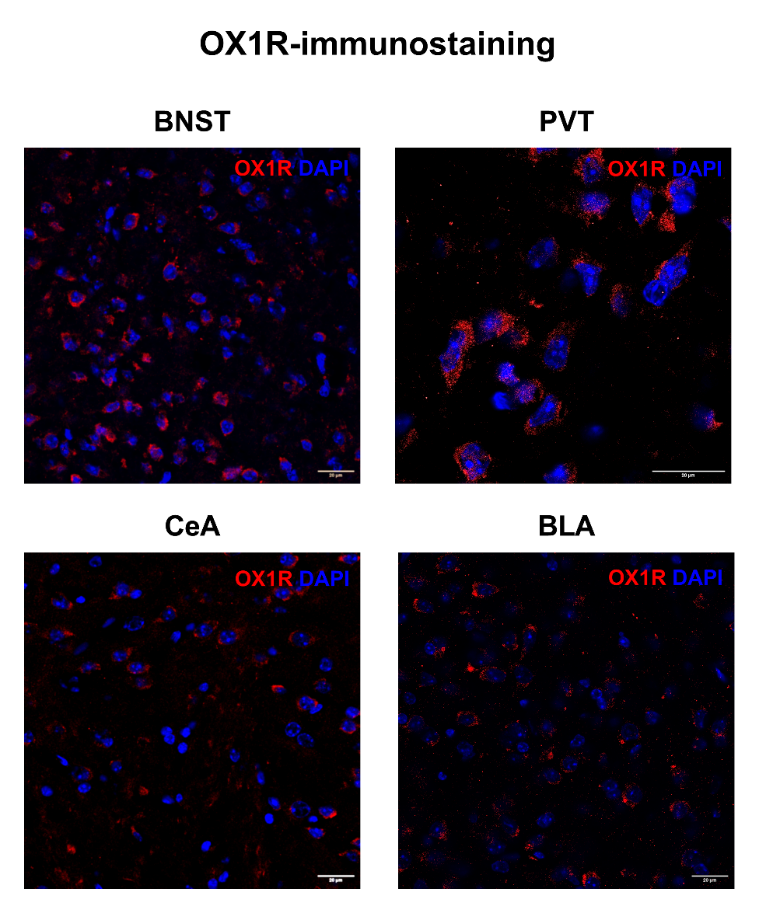


**Supplementary Fig 1:** Representative image of OX1R-immunostaining in the Bed Nucleus of Stria Terminalis (BNST), Paraventricular Thalamus (PVT), Central amygdala (CeA) and Basolateral Amygdala (BLA). Blue: DAPI staining (nuclei); red: OX1R. Scale bar: 20 µm.
